# Synovial Sarcoma of the Buccal Mucosa: A Rare Case Report

**DOI:** 10.1155/2013/938291

**Published:** 2013-05-16

**Authors:** Kumar T. S. Mahesh, Indira Annamalai Ponnuswamy, Maria Priscilla David, Peeyush Shivhare, Mahalakshmi Ikkanur Puttaranganayak, Pooja Sinha

**Affiliations:** ^1^Department of Oral Medicine and Radiology, Rajarajeswari Dental College and Hospital, Ramohalli Cross, Kumbalagodu, Mysore Road, Bangalore 560060, Karnataka, India; ^2^Department of Oral Medicine and Radiology, M. R. Ambedkar Dental College and Hospital, 1/36 Cline Road, Cooke Town, Bangalore 560005, Karnataka, India; ^3^Department of Oral Medicine and Radiology, Sri Rajiv Gandhi College of Dental Science and Hospital, Chola Nagar, R. T. Nagar, Bangalore 560032, Karnataka, India

## Abstract

Synovial sarcoma (SS) is a rare malignant neoplasm that arises most commonly in joint capsules and articular tendons, but its relationship to the synovium is not always obvious. Synovial sarcoma is a malignant soft tissue tumor representing 5.6% to 10% of all soft tissue sarcomas. They are termed SS because of their histologic resemblance to the synovium, but they rarely involve a synovial structure and are thought to arise from pluripotential mesenchymal cells. The tumor usually occurs in close association with tendon sheaths, bursae, and joint capsules, primarily in the para-articular regions of the extremities, with approximately 9% occurring in the head and neck region. Synovial sarcoma has been reported rarely in the oral cavity. We report a very rare case of Synovial sarcoma of the buccal mucosa in a 24-year-old male patient.

## 1. Introduction

Synovial sarcoma (SS) is a rare malignant neoplasm that arises most commonly in joint capsules and articular tendons, but its relationship to the synovium is not always obvious. Synovial sarcoma is a malignant soft tissue tumor representing 5.6% to 10% of all soft tissue sarcomas [1]. They are termed SS because of their histologic resemblance to the synovium, but they rarely involve a synovial structure and are thought to arise from pluripotential mesenchymal cells [2, 3].

The tumor usually occurs in close association with tendon sheaths, bursae, and joint capsules, primarily in the para-articular regions of the extremities, with approximately 9% occurring in the head and neck region. Most studies have found that the median age of SS is in the third decade and approximately 66% of the patients are male. The most common sites involved in the head and neck include the hypopharynx, postpharyngeal region, and the parapharyngeal space [4].

The origin of synovial sarcoma remains unknown, but the neoplasm is thought to arise from primitive undifferentiated pluripotential mesenchymal cells unrelated to synovial tissue [5]. Synovial sarcoma has been reported rarely in the oral cavity [6]. We report a case of synovial sarcoma of the buccal mucosa in a 24-year-old male patient.

## 2. Case Report

A 24-year-old male patient reported to the Department of Oral Medicine and Radiology, with a chief complaint of a swelling on the left side of the face since 2 years. Initially swelling was smaller in size which gradually increased to the present size, associated with difficulty in mouth opening and swallowing. There was a history of weight loss over the last few months. On general physical examination, the patient was moderately built and poorly nourished. All the vital signs were within the normal limits.

**Figure 1:**
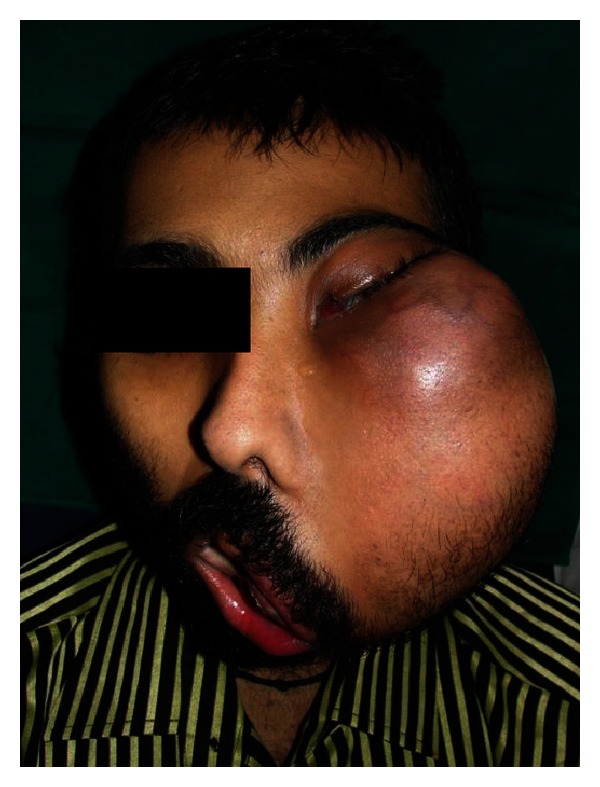
Frontal view.

**Figure 2:**
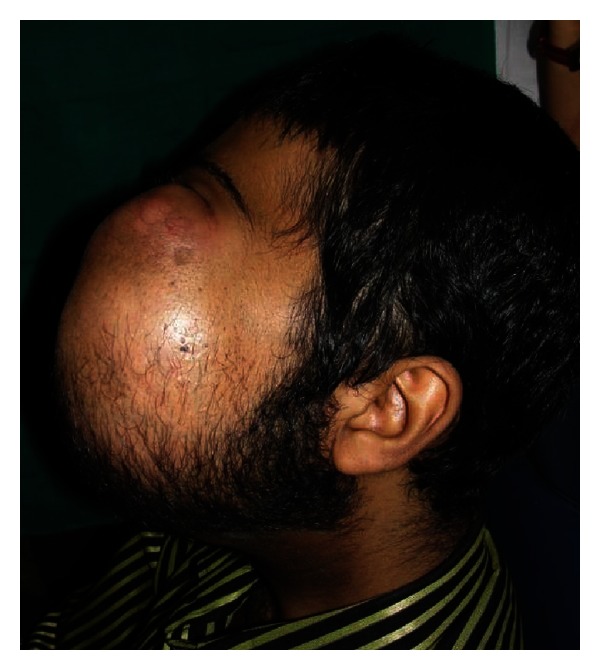
Lateral view.

Extra oral examination revealed facial asymmetry with a diffuse swelling on left side measuring 22 × 17 cms in dimension. Swelling was extending from lower eyelid superiorly to 2 cm below the lower border of the mandible inferiorly. Medially it extends from the philtrum and lateral wall of the nose to 2 cm anterior to tragus of the ear. Skin over the swelling was stretched, shiny, and erythematous. The nasolabial fold was obliterated and the patient was not able to close his mouth. Swelling was firm to hard in consistency and nontender on palpation (Figures 1 and 2).

**Figure 3:**
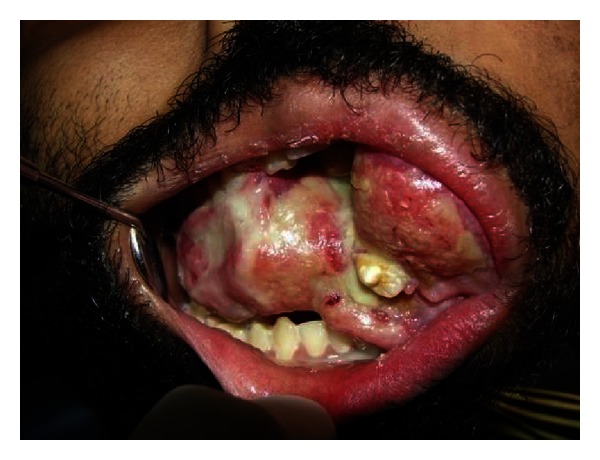
Intraoral view depicting the growth.

Intraoral examination revealed a proliferative growth measuring approximately 12 × 10 cm extending from left buccal mucosa involving upper alveolus, extending downwards crossing the midline, and extending towards the opposite side. Surface of the growth was lobulated, covered with necrotic slough, with pus discharge, and erythematous areas. The growth was firm in consistency and tender on palpation (Figure 3).

**Figure 4:**
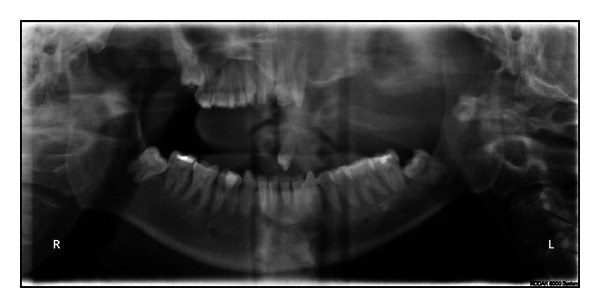
OPG showed erosion of the left maxilla and downward displacement of 24.

**Figure 5:**
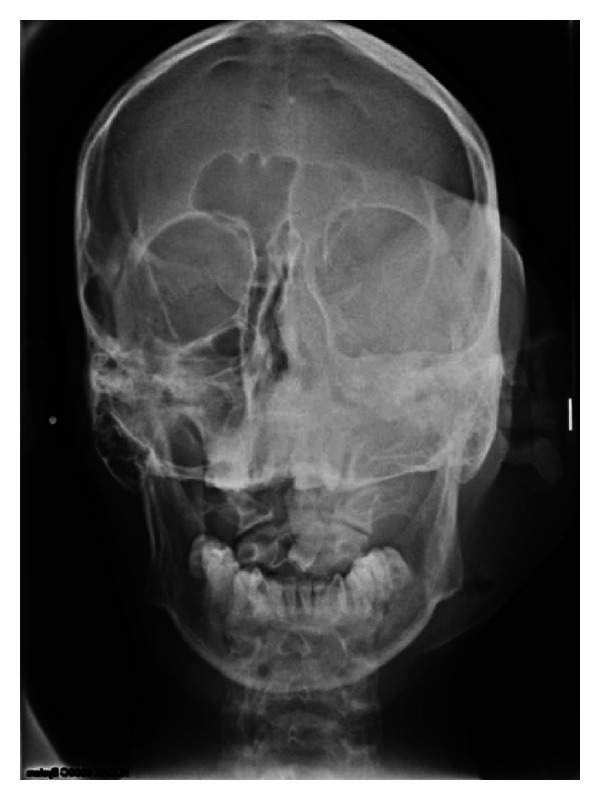
PA view showed erosion of the left maxilla and downward displacement of 24.

**Figure 6:**
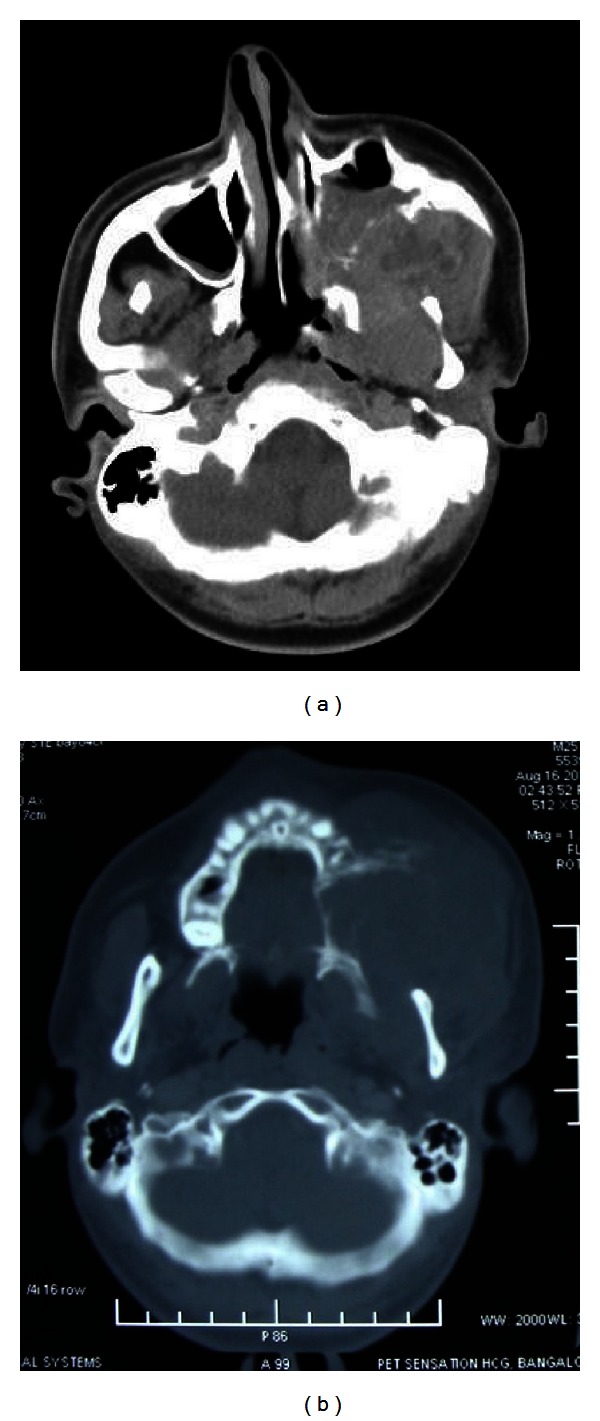
CT showing the extent of the lesion.

Investigations included complete hemogram, panoramic radiograph, skull views, CT scan, and biopsy. The hemogram showed a normal blood count with an elevated total leucocyte count and elevated ESR. Orthopantomograph and postero-anterior views showed erosion of the left maxilla and downward displacement of 24 (Figures 4 and 5). CT scan revealed a large mass epicentered over the left masticator space with destruction of the posterolateral wall of the maxillary sinus. The mass extended into the sinus cavity anteriorly, the infratemporal fossa laterally, pterygoid muscles posteriorly, and inferiorly the mass caused destruction of alveolar process of maxilla. There was destruction of superior aspect of the anterior wall of the left maxillary sinus adjacent to zygomatic arch, which also extended into orbital floor and anterolateral aspect, posteromedially extended to involve left sphenoid sinus and posterior ethmoid sinuses (Figure 6).

**Figure 7:**
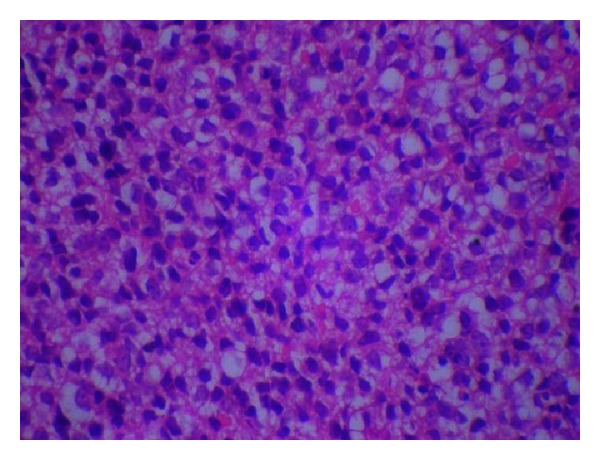
Histopathological picture showing sheets of neoplastic cells having round hyper chromatic nuclei with scanty discernible cytoplasm. Some cells have clear cytoplasm.

CT of chest revealed emphysematous changes in the lungs and irregular areas of consolidation/atelectasis in the medial aspects of both lower lobes and the inferior lingual. The mediastinal lymph nodes and bilateral axillary lymph nodes were noted and which are not significant by size criteria. Biopsy of the lesion was done under local anesthesia and on histopathological examination revealed sheets of neoplastic cells having round hyperchromatic nuclei with scanty discernible cytoplasm with some cells with clear cytoplasm suggestive of the diagnosis as poorly differentiated small round cell tumor (Figure 7).

**Figure 8:**
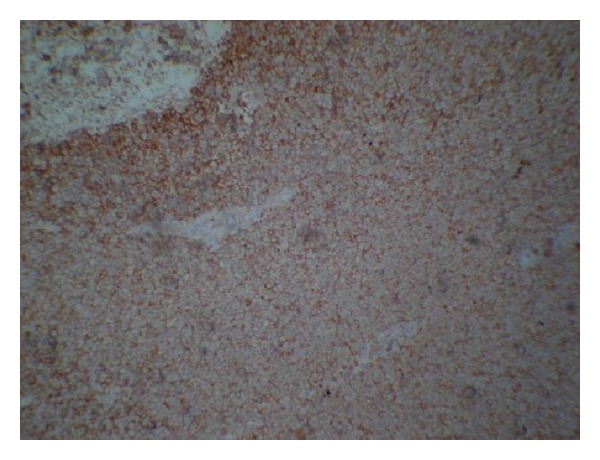
IHC revealed, tumor cells express Mic-2 with crisp cytoplasmic positivity.

Immunohistochemistry revealed the neoplastic cells expressing Vimentin, Mic-2, Bcl-2 and are negative for Myf-4, S-100, CK-7, CK-20, SMA, Desmin HMB-45, supporting diagnosis of poorly differentiated small cell variant synovial sarcoma (Figure 8).

The patient was referred to the oncologist for surgery, radiotherapy and chemotherapy. But the patient discontinued the treatment and the patient expired within a year.

## 3. Discussion

Synovial sarcoma is a well-defined clinical and morphological entity that was originally described by Simon in 1865 and was so named in 1934 by Sabrazes et al. [7]. Synovial sarcomas are a tumor of mesenchymal origin that occur predominantly in the juxta-articular regions of the lower limb; however, the cell of origin in general and of this tumor in particular is uncertain. Mainly young adults and adolescents are affected with a male-female ratio of 1.2 : 1 [8, 9]. The patient typically presents with a slow-growing palpable mass, which may grow over weeks or months, thus simulating a benign lesion [10]. More peripheral superficial lesions may be smaller, owing to earlier clinical detection. Deeper lesions might go undetected and become quite large. Lesions ranging from 2 cm to >14 cm are seen [11].

Patient with a head and neck sarcoma might present with dysphagia, hoarseness, or headache, depending on the plane of spread and site of origin of the tumor. In a large study of 672 cases, males and females were affected equally [12]. The growth was staged as stage IVB low grade using RMH Staging System [13] as follows:

 Stage IA, low grade, <5 cm;
 Stage IB, low grade, ≥5 to <10 cm, intermediate grade, <5 cm;
 Stage IIA, low grade, ≥10 to <15 cm, intermediate grade, ≥5 to <10 cm, high grade, <5 cm;
 Stage IIB, low grade, ≥15 cm, intermediate grade, ≥10 to <15 cm, high grade, ≥ 5 to <10 cm;
 Stage IIIA, intermediate grade, ≥15 cm, high grade, ≥10 to <15 cm;
 Stage IIIB, high grade, ≥15 cm;
 Stage IVA, any grade, any size, node metastases;
 Stage IVB, any grade, any size, distant metastases.

It sometimes appears in locations unrelated to synovium, and thus its origin remains unknown. There are four subtypes of Synovial sarcoma: biphasic tumors, monophasic tumors, monophasic epithelial tumors, and poorly differentiated (round cell) tumors [14, 15].

Variants of synovial sarcomas (SS) are subclassified into three groups: (i) monophasic epithelial type; (ii) monophasic spindle cell type; and (iii) biphasic type with distinct epithelial and spindle cell components. Regardless of the subtype, the conventional clues and immunohistochemical evidence of the epithelial element are characteristic of synovial sarcoma, and have led to the consensus that this tumor should be regarded as a carcinosarcoma of soft tissue origin [16]. In addition to the three subtypes, Enzinger and Weiss have described a “poorly differentiated” type of SS which shows more aggressive behavior [17].

In the present case, histopathological picture showed sheets of neoplastic cells having round hyperchromatic nuclei with scanty discernible cytoplasm. Some cells had clear cytoplasm. Immunohistochemistry revealed that tumor cells express Vimentin, Bcl-2, and Mic-2 with crisp cytoplasmic positivity suggesting the diagnosis as poorly differentiated small cell variant synovial sarcoma. Bcl-2 protein is regularly expressed in synovial sarcomas, and CD99, the product of the MIC2 gene, is seen in 67% of all cases [1].

Wide surgical resection is the primary treatment option for SS. Because complete excision of intraoral tumors is not always possible, a multimodal therapeutic approach consisting of extensive radical local excision, postoperative radiation therapy, and chemotherapy is often recommended. The metastatic potential (29.2%) and recurrence rate (20.8%) of the oral tumors appeared to be lower than those of synovial sarcoma originating at other sites [4]. Multiple recurrences were not rare. Synovial sarcoma most commonly metastasized to the lung, followed by the lymph nodes and the bone marrow [1].

Tumor recurrence typically manifested in the first 2 years after initial therapy. Prognosis is generally poor (5-year survival rate, 55%) [18]. Prognosis is adversely affected by a tumor size of more than 5 cm, tumor site, age over 60 years, high grade malignancy, and the presence of metastatic disease [9].

In the present case, the prognosis was poor; patient expired within a year after diagnosis. Synovial sarcoma is rare in the oral cavity, a feature that may increase the potential for misdiagnosis. These tumors should be considered in histopathologic differential diagnosis of both malignant primary and metastatic spindle cell tumors of the oral cavity.

The rarity of the present case is very rare malignant soft tissue tumor representing 5.6% to 10% of all soft tissue sarcomas, with approximately 9% occurring in the head and neck region, its aggressive nature, and its tendency to metastasize to other sites. This emphasizes on the early diagnosis and management of synovial sarcoma.
